# Perceptions and experiences of directly observed treatment in tuberculosis: insights from a mixed-methods cross-sectional study

**DOI:** 10.36416/1806-3756/e20240357

**Published:** 2024-12-17

**Authors:** Diana Amorim, João Pedro Ramos, Pedro Barbosa, Mariana Vieira, Raquel Duarte

**Affiliations:** 1. Serviço de Pneumologia, Unidade Local de Saúde Região de Leiria, Leiria, Portugal.; 2. EPIUnit, Instituto de Saúde Pública da Universidade do Porto, Porto, Portugal.; 3. Laboratório associado para a Investigação Integrativa e Translacional em Saúde Populacional (ITR) Porto, Portugal.; 4. Instituto de Ciencias Biomédicas Abel Salazar - ICBAS - Universidade do Porto, Porto, Portugal.; 5. Instituto Nacional de Saúde Doutor Ricardo Jorge - INSA-Porto - Porto, Portugal.

**Keywords:** Treatment adherence and compliance, Directly observed therapy, Patient satisfaction, Tuberculosis

## Abstract

**Objective:**

The demanding nature and psychosocial burdens of directly observed treatment (DOT) have opened a path to alternative strategies such as video-observed therapy (VOT), which offers comparable treatment outcomes and patient satisfaction while potentially saving time and reducing costs. The objective of this study was to evaluate the perceptions and experiences of patients and health care professionals regarding DOT and other treatment strategies implemented in Portugal.

**Methods:**

Patients with a confirmed diagnosis of tuberculosis, treated at the Vila Nova de Gaia Outpatient Tuberculosis Centre in the last two years, were asked to complete a brief questionnaire, as were health care professionals working in the northern region of Portugal. Differences were analysed with chi-square tests, complemented by thematic analysis.

**Results:**

A total of 62 individuals completed the questionnaire: 29 health care professionals and 33 patients. There were significant differences between the two groups in their views regarding the impact of DOT on treatment outcomes, with health care professionals perceiving a higher degree of negative effects and patients expressing greater satisfaction. Long travel distances, transportation issues and high costs were some of the challenges mentioned by the patients. Significant differences were also found regarding the role DOT plays in ensuring treatment adherence, with patients emphasising personal responsibility and its importance in preventing loss to follow-up and strengthening relationships with health care professionals. Dose dispensing was favoured for its convenience in specific situations, and VOT was generally preferred to reduce constant travelling. Both parties raised some concerns.

**Conclusions:**

Existing discrepancies suggest a misalignment between patient experiences and health care provider perceptions, underscoring the need for enhanced communication and a more nuanced understanding of patient perspectives when designing and implementing different tuberculosis treatment adherence strategies.

## INTRODUCTION

Tuberculosis continues to be a major global health issue, being the second leading cause of death from an infectious agent after COVID-19.^1^ Although the global incidence of tuberculosis is declining, it remains high in several countries, including Portugal, which, for 2022, reported one of the highest rates in the European Union and European Economic Area, at 14.6 cases per 100,000 population.^2^

Tuberculosis treatment involves multiple drugs taken over several months, depending on the type and extent of the disease, making it demanding because of its duration, drug load and possible adverse effects.^3^ ^,^ ^4^ Directly observed therapy (DOT), recommended by many organisations,^5^ ensures that patients take medication under supervision, helping to complete the treatment, detect adverse events early and encourage adherence.^6^ ^-^ ^8^ However, evidence on the effectiveness of DOT is mixed, some studies showing that it can hinder adherence,^6^ as well as requiring substantial clinical, human and financial resources (75% of tuberculosis treatment costs are linked to DOT).^6^ ^,^ ^8^ ^,^ ^9^ It also imposes a psychosocial burden on patients, who face daily trips to health care facilities, affecting their privacy, work life and transportation expenses, as well as increasing the stigma associated with tuberculosis, even after it has been cured.^10^ ^,^ ^11^ Alternatives to DOT are gaining traction. Although self-administered therapy (SAT) is not widely accepted, video-observed therapy (VOT) is emerging as a viable option because of advances in video technology,^12^ allowing remote monitoring of medication intake and adverse events.^13^ Studies show VOT achieves similar treatment outcomes and improves patient satisfaction while saving time and reducing costs, leading the US Centers for Disease Control to recommend it as an alternative to in-person DOT.^13^ The aim of the present study was to evaluate the perspectives of patients and of health care professionals (HCPs) on DOT and other solutions for taking medication, such as VOT or SAT with dispensing doses.

## METHODS

This was a cross-sectional study conducted in the northern region of Portugal. Our study population was composed of two subgroups of adults (individuals ≥ 18 years of age): HCPs currently working with tuberculosis in this region and patients with a confirmed diagnosis of tuberculosis who were treated at the Vila Nova de Gaia Outpatient Tuberculosis Centre in the last two years. Individuals who could not understand or complete the questionnaire were excluded.

Two different mechanisms were used for participant recruitment. The HCPs were contacted through the mailing lists of specialized services, and the questionnaire was forwarded for data collection. Patients were recruited during routine clinical appointments and were invited to provide their responses to the same questionnaire.

The questionnaire included open and closed questions to capture participant opinions and experiences based on common hindrances reported in the literature and by senior HCPs. It comprised 17 questions: some on the impact that DOT has on quality of life and some regarding views on VOT and SAT. The same questionnaire was used for both groups, with minor adjustments to personal pronouns. Google Forms was used for dissemination. Ethical approval was obtained from the Northern Regional Health Administration Ethics Committee (Reference no. CCS/2023/694), and informed consent was obtained from all participants.

Frequencies for each variable were tested using chi-square tests for independence to assess differences in perceptions between HCPs and patients. When more than 20% of the cells had expected frequencies below 5, Fisher’s exact test was conducted for 2 × 2 contingency tables,^14^ whereas the Fisher-Freeman-Halton test was applied for larger contingency tables.^15^ Statistical computations were performed with IBM SPSS Statistics software package, version 27.0 (IBM Corporation, Armonk, NY, USA). Two-tailed significance is assumed for p < 0.05. Qualitative data were analysed through thematic analysis.^16^ After familiarisation with the material, initial codes were developed and collated into potential themes. Once themes were identified, they were reviewed and refined to accurately reflect the data collected before the analysis proceeded.

## RESULTS

From September 2023 to January 2024, sixty-two valid questionnaires were completed. Twenty-nine were completed by HCPs, and thirty-three were completed by patients. Table 1 summarises the sociodemographic statistics for both subgroups. 

**Table 1:** Participant sociodemographic characteristics, by subgroup.

Characteristic	Patients	HCPs
	(n = 33)	(n = 29)
	n (%)	n (%)
Gender Female Male	8 (24.2) 25 (75.8)	23 (79.3) 6 (20.7)
Age group (years) 25-34 35-44 45-54 55-64 65-74 ≥ 75	6 (18.2) 4 (12.1) 11 (33.3) 4 (12.1) 5 (15.2) 3 (9.1)	6 (20.7) 8 (27.6) 10 (34.5) 4 (13.8) 1 (3.4) 0 (0)
Professional category Physician Nurse	n/a.	15 (51.7) 14 (48.3)

HCPs: health care professionals.

### Perceived impacts of in-person DOT

As shown in Table 2, statistical variation was found between HCPs and patients in terms of their perceptions of the negative impacts of DOT on treatment satisfaction (p < 0.001), maintenance (p < 0.001) and completion (p < 0.01). Smaller proportions of the patients reported perceived negative impacts as it relates to treatment satisfaction, maintenance and completion (66.7%, 78.8% and 78.8%, respectively), whereas the HCPs demonstrated a tendency toward greater recognition of the negative impacts, as evidenced by the higher proportions of responses in the other categories. Some variation was also registered when assessing the positive effects of DOT on the same variables, i.e., treatment maintenance (p < 0.05), completion (p < 0.05) and satisfaction (p < 0.01), particularly in the last category, the patients reporting a higher degree of satisfaction than did the HCPs (76.7% vs. 41.3%).

**Table 2:** Participant perceptions of the impact of directly observed therapy, by subgroup.

Variable	Patients	HCPs	p*
	(n = 33)	(n = 29)	
	n (%)	n (%)	
Perceived usability of DOT			
Ensuring treatment adherence Never Rarely Sometimes Often Always	4 (12.1) 3 (9.1) 0 (0) 4 (12.1) 22 (66.6)	0 (0) 1 (3.4) 0 (0) 10 (34.5) 18 (62.1)	0.047
Early detection of adverse events Never Rarely Sometimes Often Always	4 (12.1) 1 (3.0) 2 (6.1) 8 (24.2) 18 (54.6)	1 (3.4) 2 (6.9) 1 (3.4) 10 (34.5) 15 (51.7)	0.674
Negative impact of DOT			
Daily life Never Rarely Sometimes Often Always	13 (39.4) 2 (6.1) 8 (24.2) 5 (15.2) 5 (15.2)	13 (44.8) 5 (17.2) 6 (20.7) 4 (13.8) 1 (3.4)	0.426
Family and social context Never Rarely Sometimes Often Always	19 (57.6) 2 (6.1) 1 (3.0) 3 (9.1) 8 (24.2)	3 (10.3) 4 (13.8) 12 (41.4) 8 (27.6) 2 (6.9)	< 0.001
Work/study context Never Rarely Sometimes Often Always Does not work/study No response	6 (18.2) 1 (3.0) 2 (6.1) 0 (0) 4 (12.1) 19 (57.6) 1 (3.0)	4 (13.8) 3 (10.3) 9 (31.0) 9 (31.0) 4 (13.8) - -	0.025
Activities of daily living Never Rarely Sometimes Often Always	16 (48.5) 4 (12.1) 1 (3.0) 3 (9.1) 9 (27.3)	3 (10.3) 5 (17.2) 8 (27.6) 8 (27.6) 5 (17.2)	< 0.001
Overall treatment results Never Rarely Sometimes Often Always	26 (78.8) 1 (3.0) 4 (12.1) 0 (0) 2 (6.1)	18 (62.1) 6 (20.7) 4 (13.8) 1 (3.4) 0 (0)	0.068
Treatment maintenance Never Rarely Sometimes Often Always	26 (78.8) 1 (3.0) 4 (12.1) 0 (0) 2 (6.1)	11 (37.9) 12 (41.4) 5 (17.2) 1 (3.4) 0 (0)	< 0.001
Treatment completion Never Rarely Sometimes Often Always	26 (78.8) 1 (3.0) 4 (12.1) 0 (0) 2 (6.1)	13 (44.8) 10 (34.5) 5 (17.2) 1 (3.4) 0 (0)	0.002
Treatment satisfaction Never Rarely Sometimes Often Always	22 (66.7) 2 (6.1) 5 (15.2) 0 (0) 4 (12.1)	5 (17.2) 8 (27.6) 10 (34.5) 6 (20.7) 0 (0)	< 0.001
Positive impact of DOT			
Daily life Never Rarely Sometimes Often Always	7 (21.2) 3 (9.1) 7 (21.2) 6 (18.2) 10 (30.3)	2 (6.9) 3 (10.3) 7 (24.1) 9 (31) 8 (27.6)	0.520
Family and social context Never Rarely Sometimes Often Always	13 (39.4) 5 (15.2) 4 (12.1) 1 (3.0) 10 (30.3)	3 (10.3) 9 (31.0) 8 (27.6) 5 (17.2) 4 (13.8)	0.007
Work/study context Never Rarely Sometimes Often Always Does not work/study No response	8 (25.0) 0 (0) 2 (6.3 1 (3.0) 3 (9.1) 18 (54.5) 1 (3.0)	5 (17.2) 12 (41.4) 5 (17.2) 3 (10.3) 4 (13.8) - -	0.010
Activities of daily living Never Rarely Sometimes Often Always	14 (42.4) 4 (12.1) 4 (12.1) 4 (12.1) 7 (21.2)	3 (10.3) 13 (44.8) 5 (17.2) 5 (17.2) 3 (10.3)	0.008
Overall treatment results Never Rarely Sometimes Often Always No response	7 (21.2) 0 (0) 1 (3.0) 5 (15.2) 19 (57.6) 1 (3.0)	0 (0) 1 (3.4) 2 (6.9) 9 (31.0) 17 (58.6) -	0.022
Treatment maintenance Never Rarely Sometimes Often Always No response	6 (18.2) 0 (0) 2 (6.1) 5 (15.2) 20 (57.6) 1 (3.0)	0 (0) 1 (3.4) 3 (10.3) 10 (34.5) 15 (51.7) -	0.033
Treatment completion Never Rarely Sometimes Often Always No response	6 (18.2) 0 (0) 1 (3.0) 4 (12.1) 22 (66.7) 1 (3.1)	0 (0) 1 (3.4) 3 (10.3) 10 (34.5) 15 (51.7) -	0.010
Treatment satisfaction Never Rarely Sometimes Often Always No response	6 (18.2) 1 (3.0) 3 (9.1) 5 (15.2) 17 (51.5) 1 (3.0)	1 (3.4) 5 (17.2) 11 (37.9) 5 (17.2) 7 (24.1) -	0.004

HCPs: health care professionals; and DOT: directly observed therapy. *Fisher-Freeman-Halton test.

Participants mentioned several reasons that or circumstances in which a patient would take the medication without the direct observation of an HCP, namely the closure of health care services “*on weekends and public holidays*”, “*scheduled appointments at other institutions*” or “*being in hospital for cancer treatment (chemotherapy/radiotherapy) or other lengthy treatments*”. Problems such as logistics and travel to the health care centre were also mentioned, with “*logistical constraints*”, “*long distance from the outpatient tuberculosis centre*” and “*poor access to public transportation*” being the most common. Some participants also referred to professional reasons for not using DOT, such as “*extended holiday periods*”, “*starting work before the health care centres open*” and “*absence for work reasons*”, most of them agreeing upon a defined period or context. Some HCPs also mentioned a “*lack of motivation*” on the part of some patients, possibly due to a *“lack of communication*” between the two parties.

A discrepancy was also noted within subgroups, with participants acknowledging positive and negative impacts for the same variables. Despite the existing differences in the positive impact on activities of daily living (p < 0.01), work/study context (p < 0.05) and family/social context (p < 0.01); some statistical variation was also found when participants considered the negative impact in the same fields, i.e., activities of daily living (p < 0.001), work/study context (p < 0.05) and family/social context (p < 0.001). Work-related constraints, namely “*I have to be present at work, and coming here causes difficulties*” and other socio-economic factors, such as transportation issues due to “*long distances to the centre*” or “*transport network issues*” and the fact that treatment-related “*expenses are challenging*”, were some of the challenges faced by patients during DOT.

The proportion of participants who perceived DOT to have a negative impact in the social and family context was greater among the HCPs than among the patients (75.9% vs. 36.3%). When the focus was on the impact of DOT on activities of daily living, the patients mentioned a negative influence in a greater proportion than did the HCPs (39.4% vs. 13.3%). In contrast, more HCPs than patients viewed DOT as having a positive impact on activities of daily living (82.7% vs. 69.7%).

There was general agreement between HCPs and patients regarding the relationship between DOT usability and ensuring the early detection of adverse events during treatment. However, there was some disagreement regarding the effectiveness of DOT as a mechanism for ensuring adherence (p < 0.05), as fewer patients than HCPs registered a favourable perspective (21.2% vs. 3.4%), because patients understand that although DOT is assumed to provide a direct “*improvement of treatment adherence*”, “*It doesn’t make sense to make the nurse responsible for something that I should manage myself*”, given the “*work overload of health care professionals*”.

A few patients stated that without regular follow-up, “*people will end up giving up on treatment, especially as it’s a long process.*” DOT “*provides confidence to health care professionals and to the patients themselves*”. Most of the HCPs seem to perceive DOT as a positive strategy that provides “*a gain in health*”, “*complete treatment adherence and monitoring of side effects*”, “*better treatment adherence*” and “*better patient supervision, ensuring that the treatment strategy is followed correctly*”.

### Dose dispensing in SAT

Regarding the appropriateness of dispensing doses, patients and HCPs agreed that it can be appropriate (78.8% and 58.7%, respectively), for *“those who are responsible*”, as they would still take their medication in the same way. It would be an excellent way to avoid “*mobilising so many human resources*” and “*travelling*”, making it “*more convenient to take home*”. If there is *“a family member responsible for support [. . .] dispensing doses is more useful* [than is DOT]”. In regard to how SAT might work, some disagreements are noted (p<0.05), as most (54.9%) of the patients tend to think that dose dispensing could occur more than 4 times per week, whereas 35.8% of the HCPs think that it should occur 1-3 times per week, as this should not be considered routine but should only be used for “*justifiable reasons*”, such as “*weekends and public holidays*” and when “*it is impossible to go to the health centre*”. However, some HCPs stated that it can be appropriate “*to the extent that there is a balance between rigour and also reasonableness/flexibilit*y” due to “*dispensing doses being one of the facilitating measures for treatment adherence, based on a relationship of trust*.”

No statistical variation was found between patients and HCPs when they were asked if they considered SAT to be more advantageous than DOT in any aspect (see Table 3). General agreement was attained as patients and HCPs, respectively, considered SAT to be more beneficial in terms of the family and social context (69.6% and 72.4%), work/study context (72.7% and 65.5%) and activities of daily living (71.4% and 62.1%), whereas they considered it less advantageous in terms of treatment outcomes, specifically maintenance (71.4% and 69.0%) and completion (60.0% and 79.3%).

**Table 3:** Participant perceptions of the impacts of self-administered therapy and video-observed therapy, by subgroup.

Variable	Patients	HCPs	p
	(n = 33)	(n = 29)	
	n (%)	n (%)	
To what degree is...?			
Dose dispensing/SAT Inadequate Slightly adequate Moderately adequate Very adequate Extremely Adequate	5 (15.2) 2 (6) 5 (15.2) 5 (15.2) 16 (48.4)	5 (17.2) 7 (24.1) 4 (13.8) 7 (24.1) 6 (20.8)	0.107*
VOT Inadequate Slightly adequate Moderately adequate Very adequate Extremely Adequate	9 (27.3) 2 (6.1) 4 (12.1) 4 (12.1) 14 (42.4)	2 (7.0) 4 (13.8) 9 (31.0) 9 (31.0) 5 (17.2)	0.010*
At what frequency should...occur?			
Dose dispensing/SAT Never 1-3 times per week 4-6 times per week Always No response	3 (9.1) 11 (33.3) 6 (18.2) 11 (33.3) 2 (6.1)	0 (0) 17 (58.6) 6 (20.7) 2 (6.9) 4 (13.8)	0.014*
VOT Never 1-3 times per week 4-6 times per week Always No response	4 (12.1) 7 (21.2) 1 (3.0) 15 (45.4) 6 (18.2)	0 (0) 6 (20.7) 6 (20.7) 9 (31.0) 8 (27.6)	0.036*
In these contexts, is dose dispensing...than DOT?			
Family and social context Less advantageous More advantageous No response	7 (21.2) 16 (48.5) 10 (30.3)	8 (27.6) 21 (72.4) -	1.000
Work/study context Less advantageous More advantageous No response	3 (9.1) 8 (24.2) 22 (66.7)	10 (34.5) 19 (65.5) -	1.000^†^
Activities of daily living Less advantageous More advantageous No response	6 (18.2) 15 (45.4) 12 (36.4)	11 (37.9) 18 (62.1) -	0.557
Overall treatment results Less advantageous More advantageous No response	10 (30.3) 7 (21.2) 16 (48.5)	24 (82.8) 5 (17.2) -	0.093^†^
Treatment maintenance Less advantageous More advantageous No response	10 (30.3) 4 (12.1) 19 (57.6)	20 (69.0) 9 (31.0) -	0.746
Treatment completion Less advantageous More advantageous No response	9 (27.3) 6 (18.2) 18 (54.5)	23 (79.3) 6 (20.7) -	0.156^†^
Treatment satisfaction Less advantageous More advantageous No response	9 (27.3) 9 (27.3) 15 (45.4)	11 (37.9) 18 (62.1) -	0.546
In these contexts, is VOT...than DOT?			
Family and social context Less advantageous More advantageous No response	8 (24.2) 18 (54.5) 7 (21.2)	4 (13.8) 25 (86.2) -	0.192
Work/study context Less advantageous More advantageous No response	6 (18.2) 9 (27.3) 18 (54.5)	4 (13.8) 25 (86.2)	0.067
Activities of daily living Less advantageous More advantageous No response	10 (30.3) 16 (48.5) 7 (21.2)	3 (10.3) 26 (89.7) -	0.024
Overall treatment results Less advantageous More advantageous No response	9 (27.3) 9 (27.3) 15 (45.4)	17 (58.6) 12 (41.4) -	0.763
Treatment maintenance Less advantageous More advantageous No response	9 (27.3) 9 (27.3) 15 (45.4)	13 (44.9) 16 (55.2) -	0.771
Treatment completion Less advantageous More advantageous No response	9 (27.3) 9 (27.3) 15 (45.4)	14 (48.3) 15 (51.7) -	1.000
Treatment satisfaction Less advantageous More advantageous No response	8 (24.2) 12 (36.4) 13 (39.4)	5 (17.2) 24 (82.8) -	0.104

HCPs: health care professionals; SAT: self-administered therapy; VOT: video-observed therapy; and DOT: directly observed therapy. *Fisher-Freeman-Halton test. ^†^Fisher’s exact test.

### Perceived suitability of VOT

More patients than HCPs (78.8% vs. 58.7%) perceived VOT to be more appropriate than DOT as an strategy for improving adherence to tuberculosis treatment (p < 0.01), as it “*could be useful to avoid travelling*” and “*much more comfortable*”. In addition, there was a notable difference between the two subgroups in terms of the perception regarding the periodicity of VOT (p < 0.05), 55.6% of the patients favouring continuous monitoring through VOT over DOT, whereas HCPs suggested a more flexible or varied approach (Table 3). When considering the impact of VOT in major domains of life, patients and HCPs had similar perceptions of the benefits of VOT in the family and social context (69.2% and 86.2%, respectively) and in the work/study context (60.0% and 86.2%, respectively). However, more HCPs than patients (89.7% vs. 61.5%) felt that VOT is more advantageous than DOT in terms of the impact on activities of daily living (p < 0.05), especially for “*tech-savvy users who find it difficult (due to distance or daily routines) to travel to a health centre for DOT*” and with higher “*health literacy*”, patients and HCPs both stating that VOT is *“much more comfortable*”, precludes “*travelling to the outpatient centre*” and “*does not overburden the national health care system*”. Regarding treatment outcomes, neither groups appeared to have a defined notion of the impact of VOT, except in the area of treatment satisfaction, in which both perceived VOT to be more advantageous than DOT (Table 3).

Even though the advantages of VOT over DOT seem to be clear, patients and HCPs both raised some issues: “*not everyone has a mobile phone*”; “*health care centres lack an information technology platform suitable for VOT*”; *“raises privacy issues*”; *“hinders the therapeutic relationship*” and adherence due to “*forgetfulness*”; may not allow “*adverse effects* [to be] *detected at an early stage*”; and “*tech-challenged users*” may be dependent on “*someone using the equipment*” with them.

## DISCUSSION

The findings of this study provide valuable insights into the perceptions and experiences of HCPs and patients regarding different treatment adherence strategies, namely DOT, SAT and VOT. We were able to understand the perceived impact of DOT, underscoring the complexity of tuberculosis treatment adherence and highlighting the shared and divergent views of HCPs and patients across various domains, including treatment satisfaction, activities of daily living and the social context.

The HCPs and patients agreed on the relevance of DOT for the early detection of adverse events. However, the two groups were not in agreement regarding whether DOT is an adequate means of ensuring adherence to tuberculosis treatment, converging with literature that shows the rigidity of DOT to be unsupportive of treatment adherence.^6^ ^,^ ^11^ ^,^ ^17^ It is likely that HCPs perceive DOT as an effective strategy because it aligns with their clinical objectives of a rigid pattern of compliance to prevent treatment failure and the spread of drug-resistant tuberculosis strains.^18^ However, patients likely view adherence differently, considering that factors such as autonomy, convenience and their ability to manage treatment play a more significant role.^19^

Most of the study participants perceived VOT as acceptable and beneficial, although there were some disagreements regarding the frequency with which it should occur. The failure to monitor adverse events and difficulty establishing a close relationship with the HCP were major patient concerns. Although VOT offers several advantages, including convenience and potential cost savings, it also presents technological barriers and privacy concerns that must be addressed before it can effectively be implemented.^20^ Perceived barriers include limited technology skills, inadequate cellular connectivity, lack of reliable internet access, limited availability of electricity, smartphone cost and internet use fees.^21^

The physician-patient relationship, an integral part of DOT, is also a relevant dimension that supports treatment adherence. Health care providers can help patients find a less negative meaning in their tuberculosis treatment, providing support rather than mere surveillance and control.^17^ This approach may be more effective than rigidly applying DOT without considering individual factors and determinants.^17^ ^,^ ^19^ ^,^ ^22^ The barriers to observed therapy in tuberculosis treatment are multifaceted, encompassing socio-cultural, economic, technological and logistical challenges that demand a multi-agency approach with patient-centred care options.^20^ ^,^ ^21^ ^,^ ^23^

People-centred tuberculosis care involves integrating patient needs and values into treatment, providing psychosocial and socio-economic support, involving patients in treatment decisions and ensuring flexible, decentralised care models.^24^ ^-^ ^28^ It emphasises the importance of considering the social circumstances, needs and values of patients throughout their treatment journey. This approach aims to improve adherence, reduce loss to follow-up and enhance overall treatment outcomes by involving patients in their care decisions and providing holistic support.^24^ ^,^ ^25^ ^,^ ^28^

To achieve people-centred care in tuberculosis treatment, it is essential to integrate holistic support, involve patients in decision-making, provide continuous training for health care providers, implement supportive policies and address stigma and discrimination. These measures collectively enhance patient engagement, adherence and treatment outcomes, ultimately contributing to the global effort to end tuberculosis.^24^ ^-^ ^28^

Our study has some limitations. The cross-sectional design limits causal inferences, although future longitudinal studies could provide deeper insights into adherence strategies over time. Although our sample reflects experiences in northern Portugal, it was relatively small, which limits the generalisability of the results. In addition, there could have been a selection bias, as more engaged participants could lead to a positive bias. Using qualitative and quantitative methods allowed us to enhance the comprehension of the quantitative findings through thematic analysis. Furthermore, our study contributes to the currently limited body of research on subjective patient experiences in Portugal during tuberculosis treatment and DOT. The inclusion of HCPs added the comparison potentiality, offering critical insights into the intricacies of the physician-patient relationship and the current burdens associated with DOT.

In conclusion, there is an apparent misalignment between patient and HCP perspectives, highlighting a critical need for improved communication and a more person-centred approach to tuberculosis treatment. The challenges of adherence, the potential of VOT and the psychosocial burden of DOT extend beyond Portugal and have broader implications for global tuberculosis management. These findings offer valuable insights that can inform more flexible, patient-centred strategies in other countries facing similar challenges.
